# A Real-Time Energy Response Correction Method for Cs_3_Cu_2_I_5_:Tl Scintillating Dosimeter

**DOI:** 10.3390/s23218910

**Published:** 2023-11-02

**Authors:** Jiaming Li, Leilei Zhang, Jiaqi Wang, Hengyi Su, Zungang Wang, Zhiyuan Li

**Affiliations:** 1 State Key Laboratory of NBC Protection for Civilians, Academy of Military Science, Beijing 102205, China; ljiaming@stu.xjtu.edu.cn (J.L.); zll5373@163.com (L.Z.); 17713653459@163.com (J.W.); shy1850559338@163.com (H.S.); 2 Department of Nuclear Science and Technology, School of Energy and Power Engineering, Xi’an Jiaotong University, Xi’an 710049, China

**Keywords:** photon energy response, Cs_3_Cu_2_I_5_:Tl scintillation detector, dose meter

## Abstract

The uneven energy response of radiation detectors severely limits the accuracy of the dose rate meter used for radiation protection. Currently widely used in dose rate meters as a physical method of setting shielding compensation, the energy response correction error of the detector at different energies is mostly between 15 and 25%. This work designs a real-time correction method for energy response based on a novel Cs_3_Cu_2_I_5_:Tl scintillation detector to improve the accuracy of the dose rate meter used for radiation protection. The technique utilizes the idea of pulse amplitude weighting (PAW) to segment the pulse amplitude histogram. This detector achieves an almost constant energy response after our correction. The experimental results show that compared to ^137^Cs γ rays, the maximum error of the response is 8.26% in the photon energy ranging from 33 keV to 1.25 MeV, which is much better than ±30% of the recommended IEC 61526:2010, verifying the feasibility of PAW.

## 1. Introduction

In radiation protection, scintillation detectors, semiconductor detectors, and gas ionization detectors are generally used as sensing probes for dose equivalent rate meters, which are used by converting the sensors’ count rate or current response to the corresponding dose rate. The inconsistent response to photon energy, regardless of the detector, is caused by the different photon detection efficiency of the detector for other energy photons. In particular, the over-response problem is more severe in the low-energy region. It has several times the difference compared to ^137^Cs or ^60^Co and other higher energy radiation photons [1]. There is a significant error in the measurement results of the dosage rate instrument, which seriously affects the accurate monitoring of the dose rate. Therefore, its energy response correction needs to meet the measurement accuracy requirements of the dose rate instrument [2]. Several attempts have been made to solve this problem. For example, the most common method is to use physical shielding materials for correction. Most of the energy response correction errors of detectors at different energies are between 15% and 25%, which can meet the standard ±30% error requirement. However, it will reduce the detection efficiency and increase the detector’s weight. Furthermore, obtaining a more accurate dose rate in a wide energy range is not easy. We can also correct the detectors’ energy response by solving the energy spectrum-dose conversion function G(E) [3,4]. However, this method needs to solve the G(E) function, and it is complex. Usually, it requires a combination of energy spectrum measurement and offline processing, which cannot achieve real-time dose rate measurement. Moreover, the method puts higher requirements on the hardware of the dose rate measurement instrument, especially under a stronger radiation field [5,6].

In order to find a convenient and reliable calibration method, this work proposes a real-time calibration idea based on pulse amplitude weighting (PAW). The method divides the pulse amplitude generated by the energy deposited in the detector from X/γ rays into multiple intervals according to their distribution. Different intervals are given their weighting coefficients relevantly, obtained by a linear constraint algorithm, and the correction coefficient of each interval is written into the energy response real-time correction system. Finally, the response values in all intervals after correction are counted and summed. This method is different from the energy-spectrum-dose conversion G(E) function method, in which neither the acquisition of energy-spectrum data for conversion nor the high data processing capability of the instrument is required. Furthermore, the corresponding coefficients must only be written simultaneously for a particular detector. The method requires that the sensor has an energy-resolving capability. In this investigation, we used a recently developed Cs_3_Cu_2_I_5_:Tl scintillation crystal, a very effective copper-based chalcogenide material grown at the Shanghai Institute of Ceramics, Chinese Academy of Sciences. Copper(I) halide perovskite Cs_3_Cu_2_I_5_:Tl has attracted tremendous interest and has been considered an exceptionally promising scintillator due to its excellent optical properties, environmental stability, and low toxicity. The Cs_3_Cu_2_I_5_:Tl can reach a light yield of 87,000 photons/MeV under ^137^Cs γ-ray radiation and gives a remarkable energy resolution of 3.4% for 662 keV [7,8,9]. Meanwhile, a large Stokes shift of 140 nm between the PL and PLE makes the crystal have good air stability and no self-absorption. These reports suggest promise for Cs_3_Cu_2_I_5_:Tl. The 4.53 g/cm^3^ density and the effective atomic number 51.9 are competitive with other scintillators highly suitable for efficient X/γ ray detection. Therefore, it has great application value in the field of radiation monitoring [8,9]. Table 1 shows the performance comparison between Cs_3_Cu_2_I_5_:Tl single-crystal and other commonly used scintillators [10,11,12].

## 2. Methods and Principles

The scintillation detectors work by depositing energy in the scintillation body to produce a light pulse, and the magnitude of the pulse amplitude characterizes the amount of energy of the incident particles [13]. The subsequent analog circuit further amplifies and shapes the electrical signal. When a photon of energy E enters the scintillator, the detector will record a pulse signal with an amplitude of v after a series of interactions. Let the probability of interaction be p(E,v). The pulse amplitude spectral function Np(E,v) of this detector is obtained when N photons of energy E irradiate it. Without energy response correction, the area of the pulse amplitude curve is characterized as the dose rate response [14]. The real-time energy response correction technique based on PAW is mainly by weighting the counting rate partitions at different pulse amplitudes. After each interval correction, the total counting rate is characterized as the corrected dose rate response. In this way, we can correct the energy response at different energies.

Under the photon radiation field with energy E(keV) and Air *Kerma* rate ${\dot{K}}_{a}(E)$, the detector’s counting rate n(E) is divided into m clusters according to the pulse amplitude magnitude, and the system noise is filtered out by threshold screening. When the photon is irradiated into the detector, the detector will generate a pulse signal after photoelectric conversion. The amplitude of the pulse signal will be compared with a selectable threshold. This signal will be judged as valid when the pulse height exceeds this threshold, the clustering interval of the pulse amplitude will be determined, and the counter of the corresponding partition will be increased. The counting rate $n_{i}\left(E\right)$ within the partition i is also corrected and weighted by a correction factor of $k_{i}$, at which point the detector response is Equation (1):

(1) $$R_{i}\left(E\right)=\frac{\sum_{i=1}^{m}k_{i}n_{i}\left(E\right)}{{\dot{K}}_{a}(E)}$$

The detector response value before correction when m=1 and $k_{i}=1$ without impulse classification weighting is Equation (2):

(2) $$R\left(E\right)=\frac{n\left(E\right)}{{\dot{K}}_{a}}$$

Equation (1) is the corrected detector response value when m>1.

The above equation yields the energy response matrix of the detector at different energies. The value of the variance $S^{2}$ of $R_{i}\left(E\right)$ represents the magnitude of the fluctuation of the detector response at different photon energies for this detector (Equation (3)):

(3) $${\bar{R}}_{i}\left(E\right)=\frac{1}{q}\sum_{i=1}^{q}R_{i}\left(E\right),\;S^{2}=\frac{1}{q}\sum_{i=1}^{q}{\left[R_{i}\left(E\right)-{\bar{R}}_{i}\left(E\right)\right]}^{2}$$

The detector has the best energy response consistency when $S^{2}$ takes the minimum value. According to this constraint, the values of $k_{1}–k_{m}$, which are the energy response correction coefficients of the m clustering intervals, can be solved.

The minimal value of the constraint $S^{2}$, which denotes the minimum fluctuation of the detector’s response value across energies, is necessary if we wish to calculate the energy response correction factor. Therefore, it is necessary to obtain the response curve of the detector at different energy photons first. The energy points are selected using a total of ten energy points of 33 keV, 48 keV, 65 keV, 83 keV, 100 keV, 118 keV, 164 keV, 208 keV, 662 keV, and 1250 keV in IEC 61526:2010 [15].

When partitioning the pulse amplitude of the detector at different photon energies, it is known from the correction principle that the correction effect will be better if there are more partitions. However, from a practical application, the fewer the number of partitions, the better. Furthermore, the number of partitions should be divided into as few as possible to meet the energy response requirements, making the subsequent development and testing of the dose rate equipment easier. The over-response problem of the detector appears in the low-energy region, so the interval division is concentrated chiefly in the low-energy region.

The correction coefficients derived from the linear constraint algorithm of Equation (3) are written into the energy response correction software Corso v1.0 developed by LabVIEW to achieve the real-time correction of detector energy response. To evaluate the response error at a specific energy point, the energy response at that point needs to be normalized, and generally, the response value at ^137^Cs is selected for normalization, i.e., the relative energy response $R_{rel}(E)$ at a specific energy E is Equation (4):

(4) $$R_{rel}(E)=\frac{R_{i}(E)}{R(662\;\mathrm{keV})}$$

**Figure 1:**
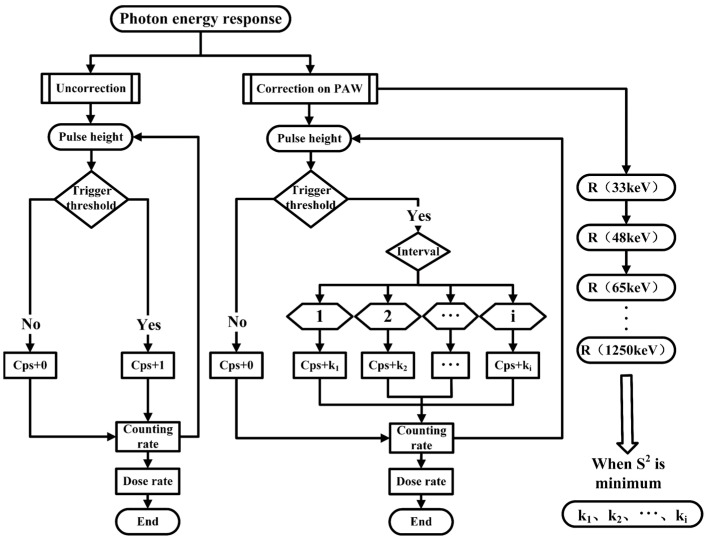
The math principle flow chart of energy response correction based on PAW.

In summary, the principle flow of the correction method of this study species is shown in Figure 1.

## 3. Experimental Setup

### 3.1. Acquisition of Energy Response Correction Coefficients

**Figure 2:**
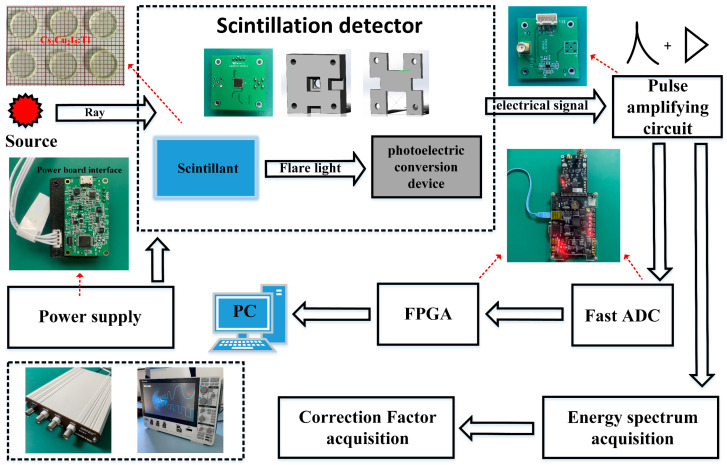
Schematic diagram of the experimental setup.

The system of radiation signal detection unit consists of a detector, signal amplification circuit, and 60 MB/s pipeline high-speed analog-to-digital converter (Fast-ADC, FADC), Field-Programmable Logic Gate Array (FPGA), and host computer software Corso v1.0. The energy spectrum acquisition system consists of a detector, a multichannel analyzer, an oscilloscope, and a PC. The schematic diagram of the experimental setup is shown in Figure 2.

The scintillation detector consists of a Cs_3_Cu_2_I_5_:Tl column scintillator of Φ 7 mm × 3 mm coupled with a Sensor-J60035 silicon photomultiplier (SiPM) with a photosensitive area of 6.07 mm × 6.07 mm. The SiPM proportionally converts the light signal generated in the scintillator into electrons and multiplies them to form an electrical pulse signal. The pulse signal output from the detector is amplified, filtered, shaped, and converted into a voltage pulse signal by a pulse signal amplifier circuit and then transmitted to the FADC for analog-to-digital conversion. The 12-bit, 60 MHz AD9238 was selected for the high-speed analog-to-digital converter. The PC software written in LabVIEW displays the counting rate in real time after the energy response correction.

**Figure 3:**
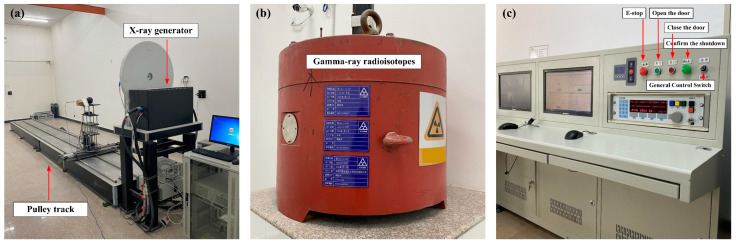
(**a**) Standard radiation field X-ray reference standard radiation device. (**b**) γ radiation source device. (**c**) Console.

The Cs_3_Cu_2_I_5_:Tl detector completed the measurement of gamma energy spectrum under ^137^Cs, ^60^Co, ^241^Am, and ^152^Eu gamma radiation sources and the measurement of X-ray energy spectrum at 33 keV, 48 keV, 65 keV, 83 keV, 100 keV, 118 keV, 164 keV, and 208 keV at the Ionizing Radiation Standard Laboratory. Since the SiPM signal is a positive pulse, we used the rising edge trigger method and set a 10 mV trigger threshold to minimize interference from noise [16]. The pulse amplitude spectra are acquired using the multichannel analyzer ORTEC EASY-MCA-8K (Easley, SC, USA). In accordance with IEC 61526:2010 requirements, the standard radiation field using radiation sources, including X-rays and radioisotopes ^137^Cs and ^60^Co, can provide certified values of Air Kerma rate from 33 keV to 1.25 MeV. The energy response experiments based on Cs_3_Cu_2_I_5_:Tl scintillation detectors in Figure 2 were done under the standard radiation field mentioned above, and the standard radiation field experimental setup is shown in Figure 3.

With reference to the requirements of IEC 61526:2010, the standard radiation field with average energy covering the range of 33 keV to 208 keV is achieved by an X-ray unit producing N40, N60, N80, N100, N120, N150, N200, and N250 narrow-spectrum radiation masses (Figure 3a), with a console (Figure 3c) to adjust the filter, tube voltage, and tube current to set the energy and dose rate of different X-rays. The standard radiation fields with average energies of 662 keV and 1.25 MeV were then realized by choosing radionuclides ^137^Cs and ^60^Co. The dose rates of different γ-rays were obtained by adjusting the source distance, and their true values were measured by a certified spherical ionization chamber. In the experiment, the detector is in charged particle equilibrium, so the effect of the particles’ Bremsstrahlung can be ignored. At this time, the value of the Air Kerma rate and the Air-absorbed dose rate is equal. The Air-absorbed dose rate is experimentally obtained from a spherical ionization chamber test, so in this work, the Air Kerma rate ${\dot{K}}_{a}$ is considered to be the same value as the Air-absorbed dose rate. The parameters of the laboratory radioactive source conditions are shown in Table 2. It should be noted that radioactive isotopes generate photons with energies of 662 keV and 1250 keV, so there are no two parameters of tube voltage and tube current.

**Figure 4:**
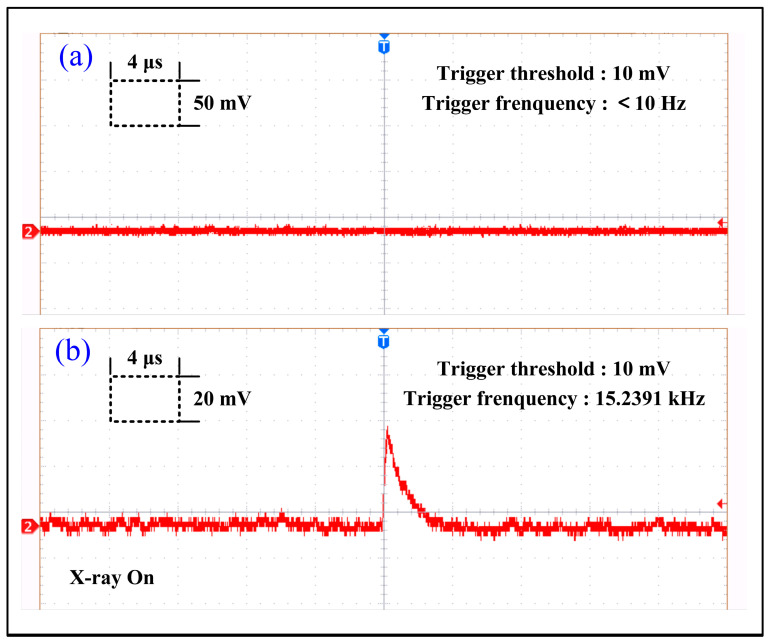
(**a**) Baseline. (**b**) Pulsed signal under photon energy radiation of 33 keV.

The detector was placed on top of the 3D motion platform, and the center of the detector was fixed at the reference point of the standard value. We tested the environmental background and system noise before the experiment in order to assess the influence of environmental background and system noise on the experimental results [17]. The detector signals were tested with the radioactive source shutter off and on and monitored in real-time by an oscilloscope. The trigger threshold was set to 10 mV to eliminate the effect of system noise as much as possible. Figure 4a shows the trigger frequency less than 10 Hz under the system noise and environmental background; Figure 4b shows the trigger frequency is above 15 kHz under the X-rays of 33 keV. In summary, the effects of environmental background and system noise on the experimental results can be ignored.

**Figure 5:**
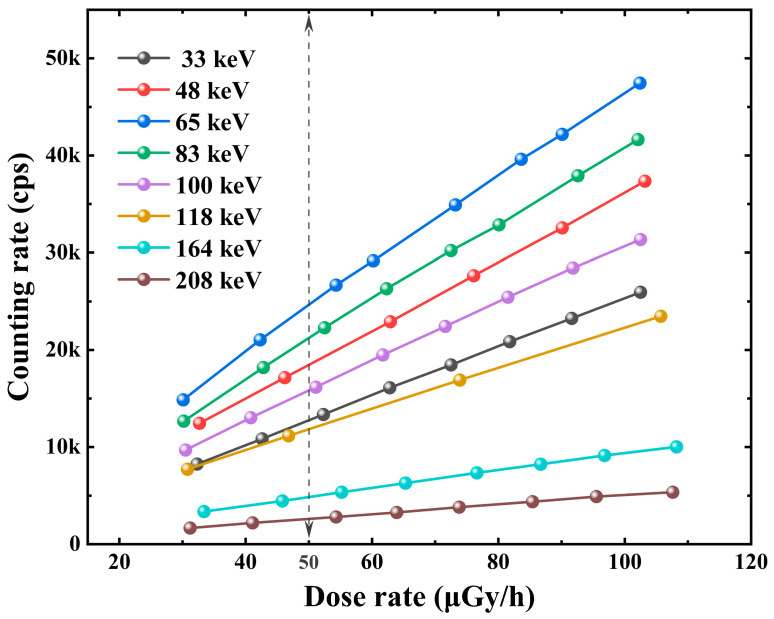
The dose rate linear response of the Cs_3_Cu_2_I_5_:Tl detector.

The acquisition energy spectrum unit consists of the multi-channel analyzer ORTEC EASY-MCA-8K and the accompanying software MAESTRO for Windows Version 7.01. To realize the signal of the received sensor, AD conversion of the pulse amplitude signal, and classification to obtain the pulse amplitude spectrum, the acquisition threshold, number of channel addresses, acquisition time, etc., can be configured within the software. To avoid the influence of the system dead time on the experimental results, the dose rate point chosen for the experiment should be within the dose rate linear response region of the detector [18]. The linear response between the dose rate and counts per second of the Cs_3_Cu_2_I_5_:Tl detector at different energies was tested by placing the detector at the reference point position of the radiation field and varying the dose rate by adjusting the magnitude of the tube current of the X-ray machine. As shown in Figure 5, the Cs_3_Cu_2_I_5_:Tl detector has reasonable linearity within about 50 μGy/h dose rate. Therefore, we chose the radiation field with a dose rate near 50 μGy/h to carry out the energy response experiment in this work.

### 3.2. Real-Time Correction of Detector Energy Response

**Figure 6:**
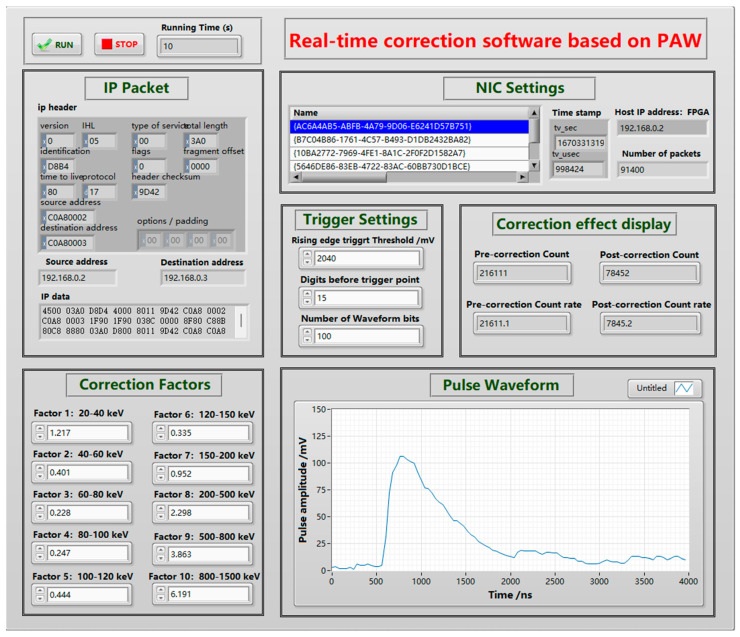
Front-end interface of LabVIEW upper computer software.

The signal acquisition part of the energy response real-time correction device uses the Xilinx Spartan6 series high-performance FPGA to process the digital waveform data output from the AD9238 and send the data to the host computer via Gigabit Ethernet. The energy response real-time calibration software was written in LabVIEW Version 2015. The program includes three modules: a digital waveform data acquisition module, a Gigabit Ethernet data splicing module, and an energy response real-time correction module. The input of the digital waveform data acquisition module was connected to the SiPM readout circuit to convert the pulse voltage signal output from the detector into digital waveform data, extract the waveform amplitude value, and transmit it to the PC. The Gigabit Ethernet data splicing module was used to transfer data from the energy response real-time correction program to the digital waveform data acquisition module to achieve real-time energy response correction. The real-time energy response correction module was used to process the shape amplitude information extracted from the digital waveform data acquisition module, perform energy response correction, and display the counts or counting rate (including the actual and corrected values) in real time. These enable digital waveform data acquisition, amplitude extraction, energy response correction factor setting, and count displaying. The front-end interface of the upper computer software is shown in Figure 6. The software allows real-time adjustment of correction coefficients, set of trigger thresholds, real-time monitoring of pulse waveform signals, and real-time display of actual and actual counting rates, corrected counts, and corrected counting rates.

## 4. Data Processing and Analysis

### 4.1. Calculation of Correction Factor

**Figure 7:**
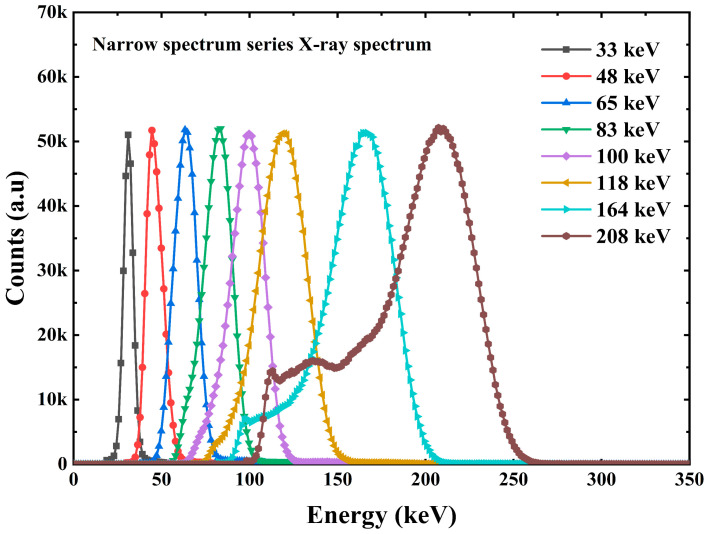
The X-ray pulse amplitude spectrum of Cs_3_Cu_2_I_5_:Tl detector.

**Figure 8:**
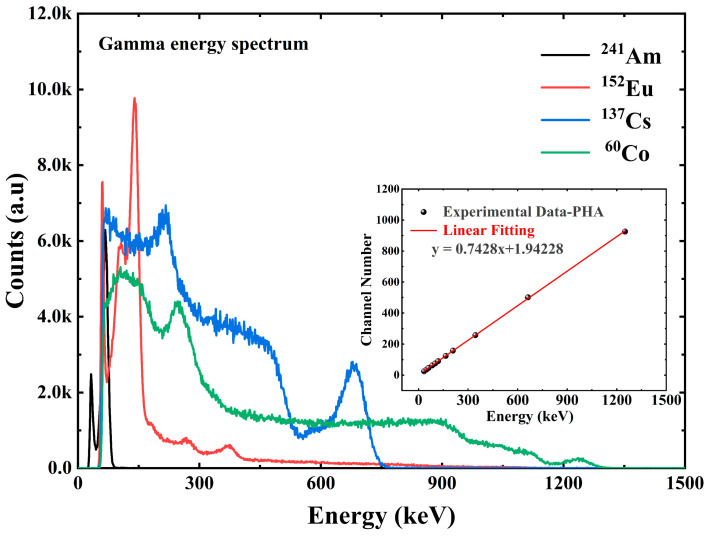
The γ pulse amplitude spectrum of Cs_3_Cu_2_I_5_:Tl detector.

The pulse amplitude spectrum acquired by the multichannel analyzer ORTEC EASY-MCA-8K is shown in Figure 7 and Figure 8. Figure 7 shows the X-ray pulse amplitude, in which we can observe that when the incident X-ray energy reaches 164 keV, the Compton flat appears in front of the X-ray energy spectrum. The interaction between X/γ ray and matter mainly includes the photoelectric effect, Compton scattering, and electron pair effect. For the same substance, the three effects have a certain dependence on the energy of the incident photon. In the case of incident low-energy photons, the photoelectric effect is dominant. As the incident photon energy increases, the Compton scattering effect gradually increases so that we can see the appearance of the platform in the front in the energy spectrum at 164 keV and 208 keV energies. For photons with energy less than 164 keV, the Compton scattering effect is so weak that we cannot see the Compton platform very clearly. Figure 8 shows the γ pulse amplitude spectrum under the γ radiation device. Figure 8 also shows the energy versus channel address scaling information obtained from the peak position information of the full energy peak, which is used to calibrate the linear relationship between channel address and photon energy in the multichannel analyzer ORTEC EASY-MCA-8K.

**Figure 9:**
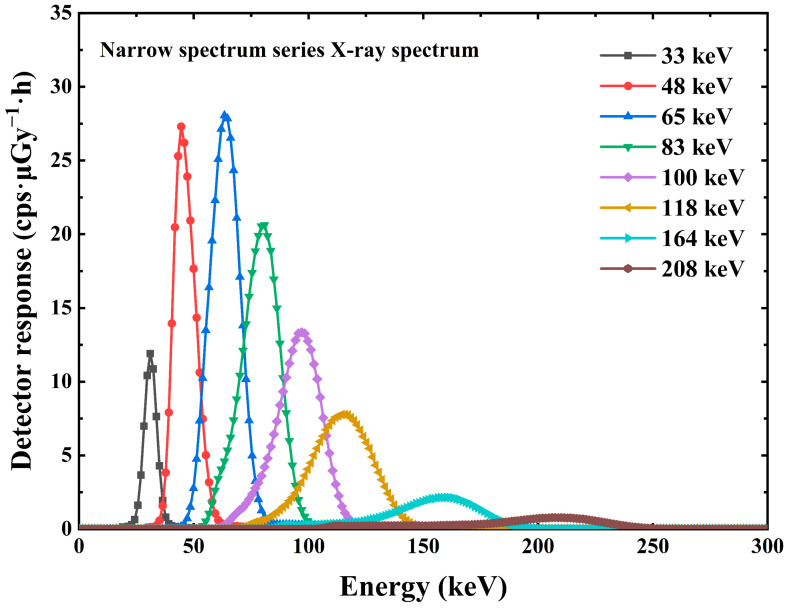
The X-ray response graph of Cs_3_Cu_2_I_5_:Tl detector.

**Figure 10:**
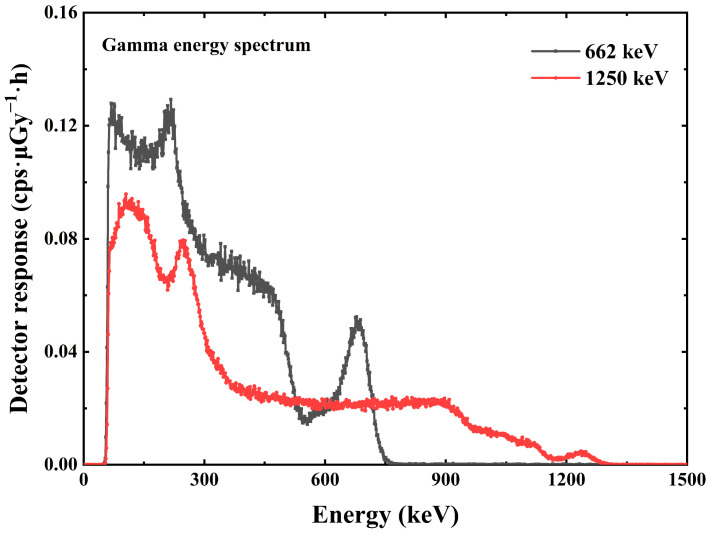
The γ response graph of Cs_3_Cu_2_I_5_:Tl detector.

The scale information between the energy and the channel address in the multichannel analyzer allows the conversion of the channel address in the horizontal coordinate to energy. The vertical coordinate in the pulse amplitude spectrum is the counts, which can be converted into the counting rate by the information of the measured spectrum time. The counting rate was divided by the dose rate at the standard position to obtain data on the detection efficiency of the detector at different energies, i.e., the detector response at different energies [19,20]. A total of 10 X/γ ray data measurements covering the energy range of 33 keV–1.25 MeV were completed for the average energy, as shown in Figure 9 and Figure 10.

The initial energy response of the detector without correction is the area of the detector response spectra at different energies. By dividing the response spectrum into multiple intervals according to the energy or pulse amplitude values, the corresponding coefficients corrected the different intervals and summed up the energy response of the detector after energy response correction. The energy spectrum information was used to classify the energy into ten intervals in this study: 20–40 keV, 40–60 keV, 60–80 keV, 80–100 keV, 100–120 keV, 120–150 keV, 150–200 keV, 200–500 keV, 500–800 keV, and 800–1500 keV, covering the photon energy range of 33 keV–1.25 MeV. Moreover, scale out the detector energy and pulse amplitude information and experimentally verify the linear relationship between incident photon energy and pulse amplitude, as shown in Table 3.

The real-time correction of the energy response based on PAW is mainly required for the convergence of the detector response values at different energies. Under this constraint, the minimum value of the variance of $R_{i}\left(E\right)$ in Equation (2) represents the stability of the detector response.

According to Equation (3), when $S^{2}$ takes the minimum value, the values of $k_{1}–k_{m}$ are obtained, which are the energy response correction coefficients for the m clustering intervals. Under the experimental conditions, this coefficient will be used as the base value and will be fine-tuned according to the actual energy response correction coefficient during the experiment. In this experiment, the pulse amplitude values of the detector were divided into ten pulse amplitude intervals, and the resulting coefficients corresponding to each interval were calculated, as shown in Table 4.

### 4.2. Conversion of Counting Rate to Dose Rate

Equation (1) can be used to obtain the response value of the detector when we have the correction coefficient for each pulse amplitude value interval. This value is used to confirm the accuracy and viability of the energy response correction result. The counting rate values at different energies can also be obtained from the correction factors and Equation (5), and the corrected counting rate can convert the actual dose rates in the radiation field.

(5) $$N_{i}\left(E\right)=\sum_{i=1}^{m}k_{i}n_{i}\left(E\right)$$

**Figure 11:**
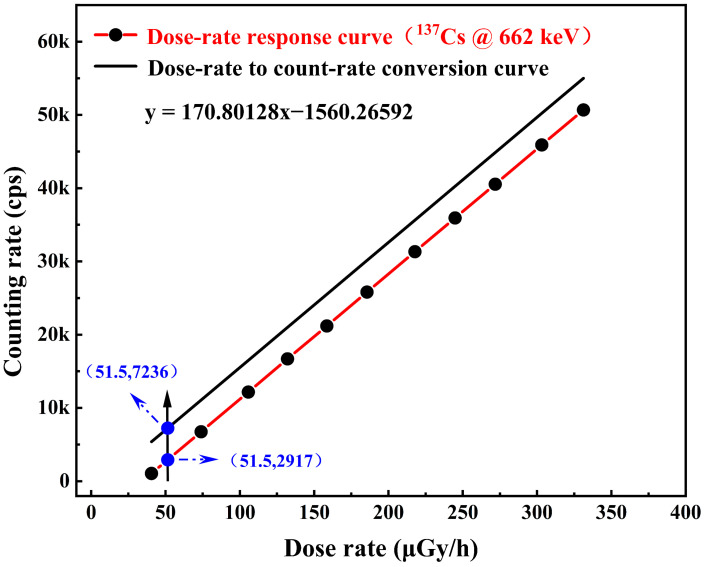
The dose-rate to count-rate conversion function graph.

The $k_{i}$ is the correction factor of interval i, and $n_{i}\left(E\right)$ is the counting rate of interval i. The total counts $N_{i}\left(E\right)$ is obtained by correcting and weighting the counting rates within all partitions. The dose-rate to count-rate conversion function is obtained by fitting and correcting the experimental data of the detector’s dose rate response at 662 keV photons. By positioning the detector at a reference point within the radiation field and varying the dose rate at the detector location by adjusting the distance between the detector and the ^137^Cs radiation source, the linear response curve of the dose rate versus counting rate of the Cs_3_Cu_2_I_5_:Tl detector at 662 keV was obtained. At this time, under the 662 keV γ photon radiation field, when the Air-absorbed dose rate is 51.5 μGy/h, the counting rate of the detector is 2917 cps. The counting rate value after correction by the PAW method is 7236 cps obtained from Equation (5), and the dose rate response curve of the detector is reset to obtain the conversion function between the counting rate and the dose rate, as shown in the Figure 11.

### 4.3. Analysis of Energy Response Correction Results

Set the correction coefficients in the energy response real-time correction upper computer software according to Table 4. When the upper computer program captured the valid nuclear pulse waveform data, it first extracted the amplitude, then used the amplitude to determine the energy interval in which the pulse falls, and finally used the set correction coefficients of the energy intervals as the pulse counts, resulting in the correction of the energy response [21], implemented by the LabVIEW program in the host computer and displayed in real time. A total of ten measurements were taken under each energy condition, and the counting rate (counts per second, cps) values in the table are the arithmetic averages of the ten measurements under each energy condition. The relative energy response was calculated from Equation (4).

**Figure 12:**
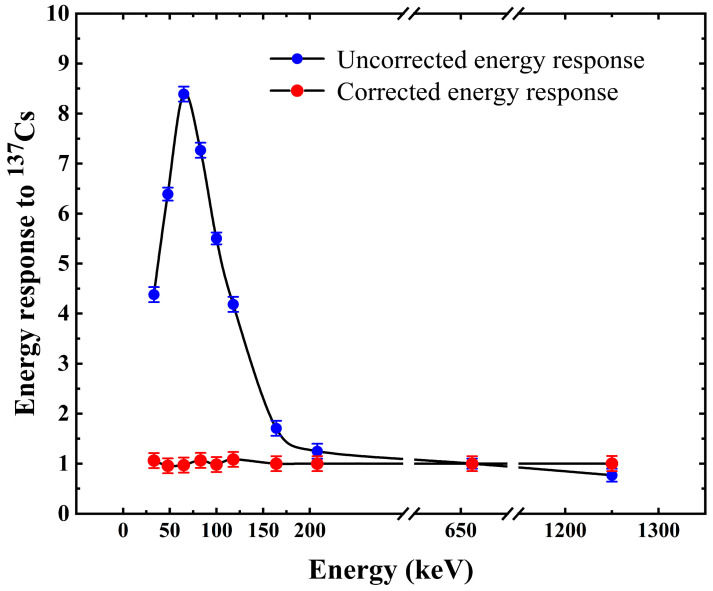
Comparison of energy response correction before and after PAW.

**Figure 13:**
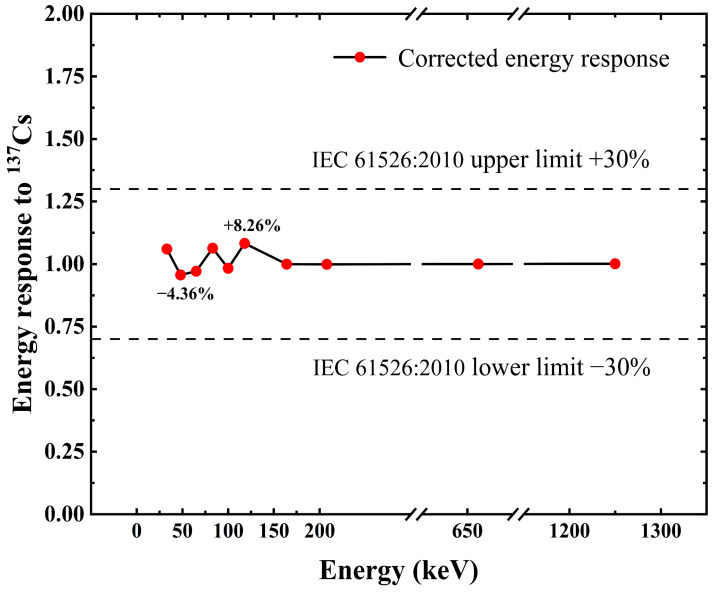
Relative response diagram after PAW of the Cs_3_Cu_2_I_5_:Tl detector.

The effect of the energy response correction on PAW is shown in Figure 12 and Figure 13. The uncorrected energy response curve in Figure 12 shows that the photon energy response of the Cs_3_Cu_2_I_5_:Tl detector before energy response correction is inconsistent in the energy range from 33 keV to 1.25 MeV. The detector has severe over-response in the low-energy area. For the Cs_3_Cu_2_I_5_:Tl detector, the response value peaks at photon incidence with an energy of 65 keV. The maximum response difference from low to high energy can be as much as eight times without photon energy response correction, which will seriously affect the accurate measurement of radiation field dose equivalent.

Figure 12 shows the detector response curves before and after energy response correction by the PAW method. Figure 13 shows the detector response error curves at different energies for the Cs_3_Cu_2_I_5_:Tl detector after energy response correction by the PAW method. Figure 13 identifies the maximum allowable error range (±30%) in the standard IEC 61526:2010, and the maximum error of the detector after energy response is 8.26% in the positive direction and 4.36% in the negative direction. The maximum error of the relative photon response of the detector after calibration is 8.26%, which is much better than the ±30% requirement of IEC 61526:2010.

**Figure 14:**
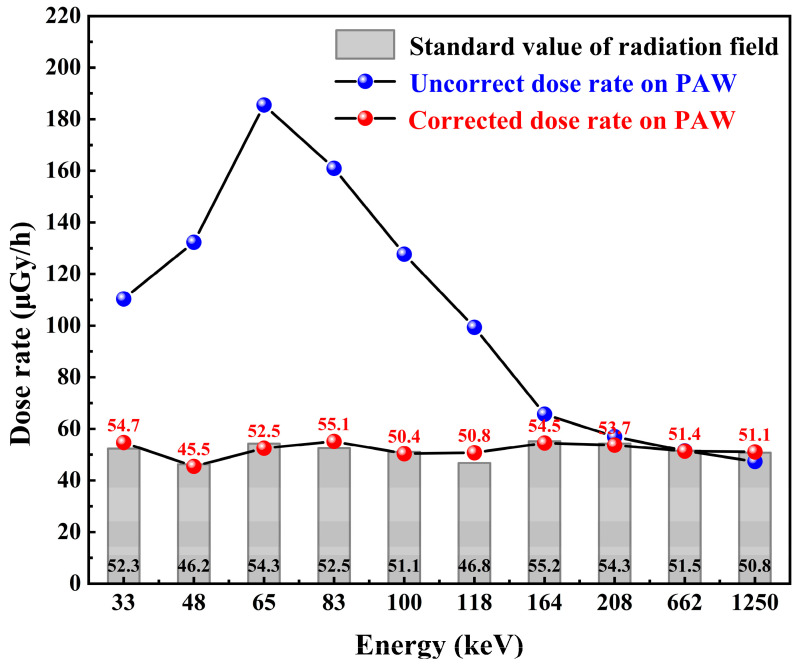
Comparison of standard and measured dose rate.

**Figure 15:**
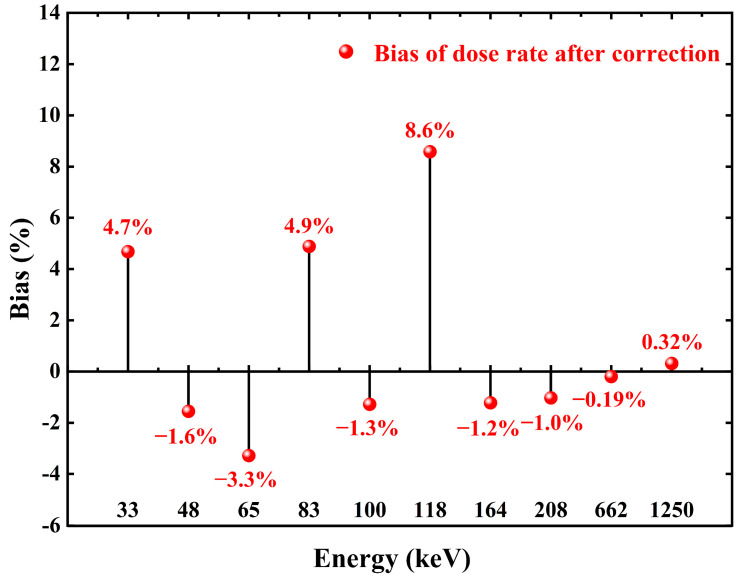
Plot of relative bias dose rate.

After the energy response correction, regardless of the energy of the incident photon, the count rate of the detector in the radiation field with different energy photons but the same dose rate is equal. That is, the count rate displayed by the detector is linearly related to the dose rate in the radiation field, regardless of the energy, which is the purpose of the PAW method. In this way, we can easily carry out the conversion between the counting and dose rates. There is no need to worry about the measurement error caused by the inconsistent detection efficiency of the detector for different energy photons. Dose rate conversion before and after correction can be performed using the conversion function in Figure 11. Figure 14 shows the measured values before and after correction against the standard values. The relative deviation of the measured dose rate from the radiation field standard values after correction by the PAW method is shown in Figure 15, and the relative deviation of the dose rate is calculated to be in the range of −3.3% to 8.6%.

## 5. Conclusions

This paper proposes an example of a real-time correction technique of energy response based on pulse amplitude weighting (PAW) for Cs_3_Cu_2_I_5_:Tl scintillation detectors. The correction of photon energy response in the energy range of 33 keV–1.25 MeV is achieved by zonal correction of the pulse amplitude of the detector, and the principle and method of photon energy response correction based on PAW are introduced. The related characteristics of the standard radiation field are described. The experimental results show that with the use of the PAW method to correct the photon energy response of the Cs_3_Cu_2_I_5_:Tl scintillation detector, the response is almost constant between the different photon energies and the difference only ±8.26% relative to ^137^Cs. The detector satisfies the energy response specifications specified by the IEC 61526:2010 standard [15] at various energies in relation to ^137^Cs. For detectors that adhere to the IEC 61526:2010’s energy response specifications, the energy response error is within 30%. The error range of the dose rate readings is −3.3% to 8.6% after energy response correction and dose rate conversion. By digitally adjusting the energy response of the detector in the dose rate meter in real time, the accuracy of dose rate meter measurements at various photon energies is increased. The method has fewer energy response correction coefficients, and all correction coefficients can be obtained simultaneously instead of identifying the energy within the radiation field each time. Analog devices such as comparators can be used for the microcontroller to achieve the energy response correction of the detector, thus reducing the development cost of the dose rate meter, which has the value of promoting the application in the radiation environment dose monitoring system.

## Figures and Tables

**Table 1:** The performance comparison between Cs_3_Cu_2_I_5_:Tl single-crystal and other commonly used scintillators.

Performance Indicators	NaI:Tl	SrI_2_:Eu	LaBr_3_:Ce	Cs_3_Cu_2_I_5_:Tl
Light yield (^137^Cs, Photons/MeV)	38,000	>90,000	70,000	87,000
Energy resolution (^137^Cs)	6%	2.6%	2.6%	3.4%
Density (g/cm^3^)	~3.67	~4.55	~5.29	~4.53
Effective atomic number	50	50.2	48.3	51.9
Deliquescence	yes	yes	yes	no
Self-absorption	no	weak	yes	no
Cost	high	high	very high	middle

**Table 2:** Experimental parameters of radiation field.

Energy (keV)	Tube Voltage (kV)	Tube Current (mA)	Dose Rate (μGy/h)
33	40	0.09	52.3
48	60	0.09	46.2
65	80	0.17	54.3
83	100	0.26	52.5
100	120	0.27	51.1
118	150	0.18	46.8
164	200	0.28	55.2
208	250	0.34	54.3
662	-	-	51.5
1250	-	-	50.8

**Table 3:** Correspondence between energy and pulse amplitude.

Energy (keV)	Pulse Height (mV)	Energy (keV)	Pulse Height (mV)
0	0	120	58.54
20	9.76	150	73.17
40	19.51	200	97.56
60	29.27	500	243.9
80	39.02	800	390.2
100	48.78	1500	731.7

**Table 4:** Energy response correction intervals and correction factors.

Energy Interval (keV)	Correction Factor (a.u.)	Energy Interval (keV)	Correction Factor (a.u.)
20–40	1.217	120–150	0.335
40–60	0.401	150–200	0.952
60–80	0.228	200–500	2.298
80–100	0.247	500–800	3.863
100–120	0.444	800–1500	6.191

## Data Availability

No new data were created or analyzed in this study. Data sharing is not applicable to this article. There was no public involvement in any aspect of this research.
